# Optimization of ultrasonic-assisted extraction of total flavonoids from *Oxalis corniculata* by a hybrid response surface methodology-artificial neural network-genetic algorithm (RSM-ANN-GA) approach, coupled with an assessment of antioxidant activities†

**DOI:** 10.1039/d4ra05077k

**Published:** 2024-12-10

**Authors:** Deng-Zhao Jiang, Dan-Ping Yu, Ming Zeng, Wen-Bo Liu, Dong-Lin Li, Ke-Yue Liu

**Affiliations:** a School of Pharmacy and Life Science, Jiujiang University Jiujiang 332005 China jiangdengzhao@126.com; b Jiujiang Key Laboratory for the Development and Utilization of Traditional Chinese Medicine Resources in Northwest Jiangxi Jiujiang 332005 China; c Analytical and Testing Center, Jiujiang University Jiujiang 332005 China

## Abstract

The objective of this research endeavor is to refine the ultrasonic-assisted extraction technique for total flavonoids from *Oxalis corniculata* (TFO), utilizing a synergistic approach combining response surface methodology (RSM) and artificial neural network integrated with genetic algorithm (RSM-ANN-GA). The optimized extraction parameters determined through RSM yielded a TFO concentration of 13.538 mg g^−1^ under the following conditions: an ethanol concentration of 61.95%, a liquid–solid ratio of 41.06 mL g^−1^, an ultrasonic power setting of 351.57 W, and an ultrasonic exposure duration of 58.95 minutes. Conversely, the RSM-ANN-GA approach identified an even more refined set of conditions, achieving a TFO concentration of 13.7844 mg g^−1^, with an ethanol concentration of 58.93%, a liquid–solid ratio of 41.16 mL g^−1^, an ultrasonic power of 350.22 W, and an ultrasonic exposure time of 58.18 minutes. These findings underscore the superior predictive accuracy and enhanced extraction efficiency offered by the RSM-ANN-GA model over the conventional RSM method. Furthermore, the study demonstrated that TFO possesses a potent antioxidant effect, as evidenced by its ability to scavenge DPPH, hydroxyl, and superoxide anion free radicals *in vitro*, highlighting its potential as a valuable source of natural antioxidants.

## Abstract 2

This study aims to optimize the UAE of TFO through an integrated methodological framework combining RSM-ANN-GA. The synergistic application of these techniques is intended to improve the precision and efficiency of the extraction process.

## Graphical abstract

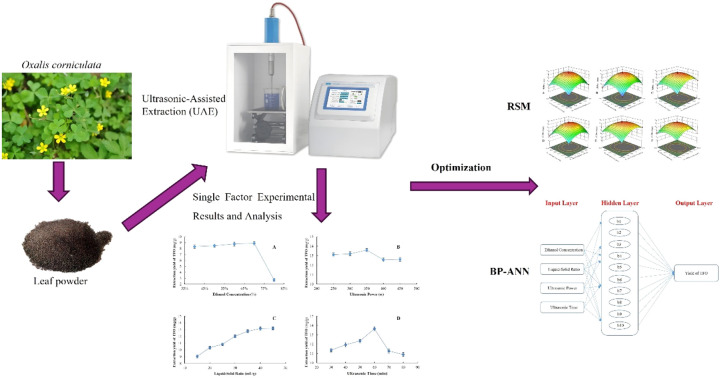

## 1 Introduction

*Oxalis corniculata*, a globally widespread member of the Oxalidaceae family, is prevalent in Asia, South Africa, and South America. Known as an invasive yet medicinally potent weed, it has been valued in traditional medicine in China, Pakistan, and India.^1^ Recent studies have reported various pharmacological activities, including anticancer, antibacterial, antifungal, insecticidal, and antioxidant effects, *etc.*^2–12^ Phytochemical analysis reveals the presence of alkaloids, flavonoids, terpenoids, glycosides, and saponin, *etc.*^6–8,12–14^ Among these, flavonoids stand out for their strong antioxidant and antimicrobial properties. These bioactive compounds make *O. corniculata* a valuable resource for advancing modern pharmacology, nutraceutical development, and cosmeceutical formulations.^5,13^

Conventional extraction methods for flavonoids, such as percolation and decoction, require significant solvent and energy. Ultrasound-assisted extraction (UAE) is a more efficient and sustainable alternative. UAE uses ultrasonic waves to create acoustic cavitation, which disrupts cellular structures and enhances the release and solubilization of target compounds. This improves extraction efficiency and reduces environmental impact. UAE accelerates the leaching of bioactive compounds, boosting extraction efficacy while minimizing resource consumption. Therefore, UAE has become widely adopted for extracting bioactive plant components.^15–19^

The efficacy of component extraction depends on factors such as solvent concentration, extraction duration, ultrasonic power, pH levels, and temperature, *etc.* The complex nature of botanical constituents often results in a non-linear extraction process. Despite its inefficiency and resource wastage, the one-variable-at-a-time (OVAT) method is still widely used for its simplicity and intuitiveness. However, OVAT fails to detect interactions between factors and lacks a comprehensive understanding of all determinants.^20^

In contrast, the design of experiments (DoE) approach offers significant advantages by requiring fewer experiments and lower costs.^21^ Orthogonal design, a traditional DoE method, investigates the impact of different parameters through a limited number of experimental runs. When used correctly, orthogonal design effectively determines the optimal combination of factor levels, making it a valuable tool for optimizing extraction processes.^21^

Novel optimization methodologies, such as Response Surface Methodology (RSM) and Artificial Neural Networks (ANN), have gained attention for extracting plant-based medicinal ingredients. RSM, a statistical technique, models complex processes and optimizes parameter interactions.^22,23^ ANNs add computational intelligence by recognizing intricate patterns and making sophisticated predictions.^24–27^ Compared to RSM, the integration of RSM, ANN, and Genetic Algorithms (GA)—known as RSM-ANN-GA—demonstrates superior proficiency in analyzing observational data and creating predictive models for complex, nonlinear processes. This combination has evolved into a more powerful and accurate optimization tool.^25,27–31^

Current literature shows a notable lack of research on enhancing the extraction of TFO, particularly using ANN methodologies. To address this, we first employed RSM to optimize the TFO extraction process. Next, an ANN model was developed using the experimental data from RSM design points. Finally, the optimal extraction conditions were determined using a synergistic RSM-ANN-GA approach, followed by *in vitro* assessments of the antioxidant activities of TFO.

## 2 Results and discussion

### 2.1. Standard curve

**Fig. 1:**
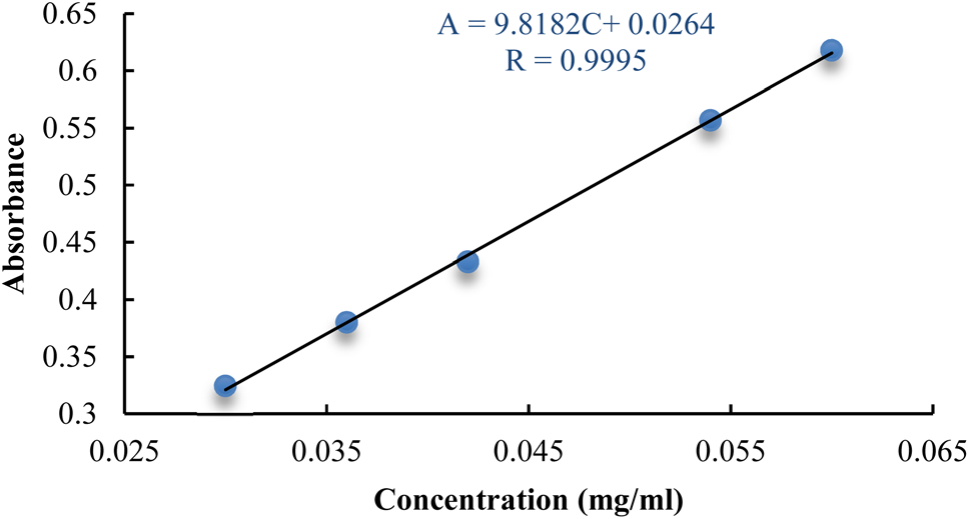
The standard curve of rutin.

The deduced linear regression equation manifests as: *A* = 9.8182*C* + 0.0264, complemented by a correlation coefficient *R* = 0.9995. Such results unequivocally validate that, within the concentration domain spanning from 0.03 to 0.06 mg mL^−1^, the concentration of rutin bears an exceptional linear relationship with absorbance. This finding attests to the precision and steadfast reliability of the adopted measurement method (Fig. 1).

### 2.2. Identification and characterization of TFO

**Fig. 2:**
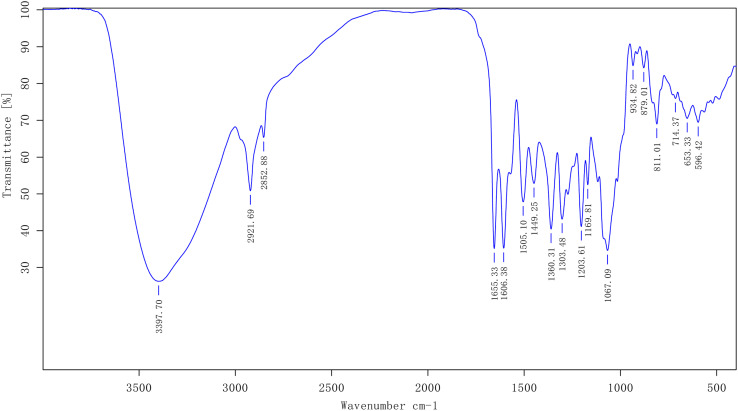
FTIR spectrum analysis of the extraction obtained by UAE.

Following the interaction between the extraction solution and magnesium hydrochloride powder, a notable change occurred, manifesting as a deep purple coloration, which unequivocally signals the inclusion of flavonoids in the extracted mixture. The FTIR spectral profile for the extract derived from UAE is delineated in Fig. 2, revealing the molecular vibrations characteristic of the constituents within the extract.

The broad and intense absorption band observed around 37.70 cm^−1^ is likely attributable to the stretching vibrations of the O–H group, which typically occur within the range of 3600–3200 cm^−1^.

An absorption peak at 2921.69 cm^−1^ could be attributed to the stretching vibrations of both aliphatic and aromatic C–H groups, which are commonly observed in the range of 2950–2850 cm^−1^. Meanwhile, the peak at approximately 1655.33 cm^−1^ may be indicative of C=O stretching vibrations, typically found within the spectral window of 1680–1620 cm^−1^. Peaks located at 1606.38 cm^−1^ and 1449.25 cm^−1^ might be associated with the skeletal vibrations of the benzene ring, while the feature at 1067.09 cm^−1^ is likely due to the deformation vibrations of C–C bonds. All the identified absorptions, when considered in concert, point unmistakably toward the existence of flavonoids, their distinctive spectral signatures weaving a tale of the sample's chemical composition.^15,32^

### 2.3. Single factor experimental results and analysis

#### 2.3.1. Effect of ethanol concentration

**Fig. 3:**
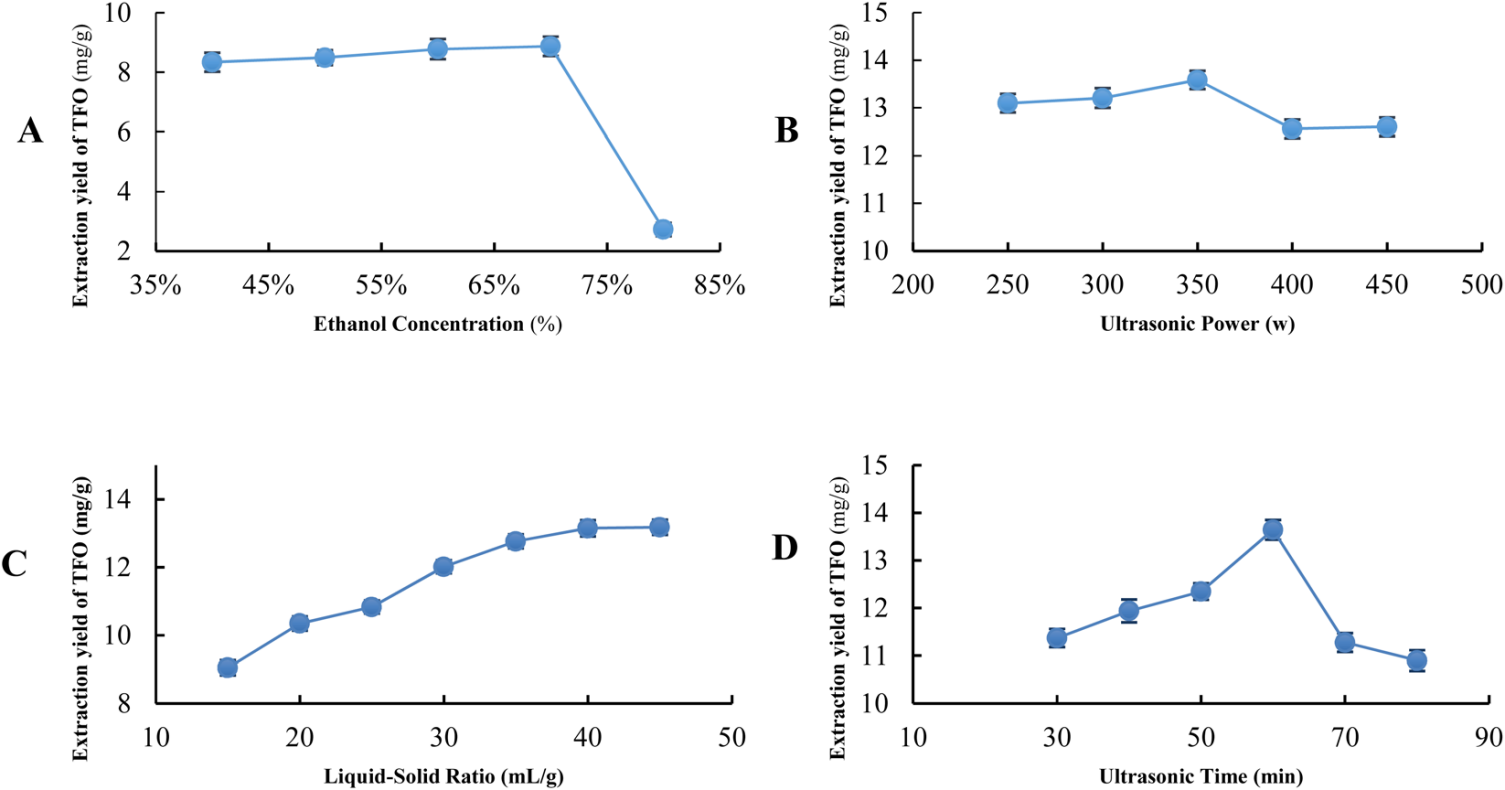
Effect on the extraction yield of TFO ((A) ethanol concentration; (B) ultrasonic power; (C) liquid–solid ratio; (D) ultrasonic time).

As illustrated in Fig. 3A, the yield of TFO extraction increases with the elevation in ethanol concentration, attributed to the reduction in solution polarity, which helps enhance the solubility of flavonoids.^33^ Notably, this increase is more pronounced between concentrations of 40% and 60%, after which the rate of increase tapers off gradually beyond the 60% mark. Upon reaching an ethanol concentration of 80%, a notable transformation occurred in the extraction solution, shifting its hue from a standard orange-yellow to a lighter shade of green. This change in coloration suggests a significant extraction of chlorophyll by the 80% ethanol, leading to a decrease in the concentration of TFO. The resultant light green tint of the solution is indicative of the elevated chlorophyll content, illustrating the complex interplay between solvent strength and the selective extraction of plant compounds.

#### 2.3.2. Effect of ultrasonic power

As illustrated in Fig. 3B, the yield of TFO extraction ascends to a zenith at 350 W concurrent with the augmentation of ultrasonic power. The ultrasonic cavitation phenomenon, characterized by the formation and subsequent implosion of bubbles, significantly enhances the diffusion of the solute and exerts a disruptive influence upon the raw materials. This dual action facilitates a higher permeability of the solvent into the matrix, thereby augmenting the extraction yield.^34^ Upon transcending the 350 W threshold, a progressive enhancement in ultrasonic power engenders a decremental shift in the extraction efficacy of TFO. This observation may be attributed to the production of hydroxyl radicals at high ultrasonic power, which can react with phenolic compounds and cause their degradation.^35^

#### 2.3.3. Effect of liquid–solid ratio

As depicted in Fig. 3C, the extraction yield of TFO ascends progressively in tandem with the augmentation of the liquid–solid ratio, achieving its apex at a ratio of 40 mL g^−1^. The effect of the solvent to material ratio can be attributed to the possibility that the reduced mixture density, resulting from a higher solvent to material ratio, increases the ultrasound wave propagation speed, reduces the attenuation of ultrasound power, and enhances the transfer of energy/distance/time.^36^

#### 2.3.4. Influence of ultrasonic time

As illustrated in Fig. 3D, in the initial stage, the extraction yield of total flavonoids (TFO) rapidly increases with the extension of ultrasonic exposure time, reaching a peak at 60 minutes. This may be due to the higher slope of the gradient solvent and the cavitation, thermal, and physical effects generated at the sample surface.^37^ Nonetheless, a paradoxical diminution in yield becomes apparent upon further extension of the ultrasonic treatment beyond this optimal time frame. The plausible rationale behind this phenomenon may be attributed to the prolongation of ultrasonic exposure, which leading to the degradation of flavonoids.^38^

In accordance with the empirical findings delineated herein, the factorial design encompasses three hierarchical levels for each of the four investigated parameters. Specifically, the ethanol concentration (*X*_1_) spans a spectrum from 50% to 60% and 75%; the ultrasonic power (*X*_2_) is delineated at 300 W, 350 W and 400 W; the liquid–solid ratio (*X*_3_) is calibrated at 35, 40 and 45 mL g^−1^; and the extraction time (*X*_4_) is set at intervals of 50, 60 and 70 minutes, as meticulously tabulated in Table S1.†

### 2.4. Optimization of extraction parameters utilizing RSM

The Box–Behnken design with RSM and ANN results were shown in Table S2.† Utilizing the sophisticated capabilities of Design-Expert 10.0 software, an exhaustive analysis of variance (ANOVA) was conducted on the dataset amalgamating both empirical and projected values, as outlined in Table 1. Adopting the extraction yield of TFO (*Y*) as the focal response parameter, the implementation of a quadratic regression analytical framework resulted in the derivation of a comprehensive quadratic multinomial regression equation. This equation elucidates the intricate interplay between the extraction yield of TFO (*Y*) and the quartet of independent variables: ethanol concentration (*X*_1_), liquid–solid ratio (*X*_2_), ultrasonic power (*X*_3_), and ultrasonic time (*X*_4_):*Y* = +13.41 + 0.65*X*_1_ + 0.53*X*_2_ + 0.094*X*_3_ − 0.096*X*_4_ + 0.22*X*_1_*X*_2_ − 0.098*X*_1_*X*_3_ + 0.24*X*_1_*X*_4_ + 0.19*X*_2_*X*_3_ − 0.50*X*_2_*X*_4_ − 0.030*X*_3_*X*_4_ − 1.84*X*_1_^2^ − 1.48*X*_2_^2^ − 1.90*X*_3_^2^ − 1.19*X*_4_^2^

**Table 1:** ANOVA for response surface quadratic model

Source	Sum of squares	df	Mean square	*F* value	*p*-Value Prob > *F*	
Model	55.59	14	3.97	42.60	<0.0001	Significant
*X*_1_-ethanol concentration	5.10	1	5.10	54.66	<0.0001	Significant
*X*_2_-liquid–solid ratio	3.31	1	3.31	35.48	<0.0001	Significant
*X*_3_-ultrasonic power	0.11	1	0.11	1.14	0.3034	
*X*_4_-ultrasonic time	0.11	1	0.11	1.18	0.2953	
*X*_1_ *X*_2_	0.20	1	0.20	2.12	0.1671	
*X*_1_ *X*_3_	0.04	1	0.04	0.41	0.5334	
*X*_1_ *X*_4_	0.24	1	0.24	2.58	0.1308	
*X*_2_ *X*_3_	0.14	1	0.14	1.55	0.2337	
*X*_2_ *X*_4_	1.01	1	1.01	10.83	0.0054	Significant
*X*_3_ *X*_4_	0.0036	1	0.0036	0.04	0.8470	
*X*_1_^2^	22.03	1	22.03	236.36	<0.0001	Significant
*X*_2_^2^	14.27	1	14.27	153.04	<0.0001	Significant
*X*_3_^2^	23.52	1	23.52	252.33	<0.0001	Significant
*X*_4_^2^	9.25	1	9.25	99.25	<0.0001	Significant
Residual	1.31	14	0.09			
Lack of fit	1.08	10	0.11	1.91	0.2783	Not significant
Pure error	0.23	4	0.06			
*R* ^2^						0.9771
Adj. *R*^2^						0.9541
Pred. *R*^2^						0.8845

An inspection of Table 1 reveals that the regression model, marked by a *p*-value less than 0.001, underscores the exceptional significance of the regression equation model, signifying an optimally efficacious fit. The insignificance of the lack of fit test (*p* > 0.05) corroborates the veracity of the quadratic polynomial regression model, substantiating its appropriateness for the dataset at hand. The concordance between the coefficient of determination (*R*^2^ = 0.9771) and the adjusted determination coefficient (adj. *R*^2^ = 0.9541) is indicative of a well-calibrated model, underscoring the reliability of predictions derived from this regression equation.^27^ An *F*-value of 1.91 for the lack of fit suggests that any discrepancy between the model's predictions and the actual observations is not statistically significant relative to the intrinsic variability (pure error). This finding implies that there is a 27.83% probability that an *F*-value of this magnitude could arise solely from random fluctuations or noise.

In the significance testing of the coefficients for the quadratic model regression equation, the Prob > *F* values for *X*_1_ (ethanol concentration) and *X*_2_ (liquid-to-solid ratio) were both lower than 0.01, indicating a highly significant linear effect on the TFO ultrasonic extraction efficiency. The hierarchy of factors impacting the extraction yield of TFO is delineated as follows: *X*_1_ (ethanol concentration) > *X*_2_ (liquid–solid ratio) > *X*_4_ (ultrasonic time) > *X*_3_ (ultrasonic power). The Prob > *F* values for *X*_1_^2^, *X*_2_^2^, *X*_3_^2^ and *X*_4_^2^ were less than 0.01, indicating a highly significant curvilinear effect on the TFO ultrasonic extraction efficiency.

**Fig. 4:**
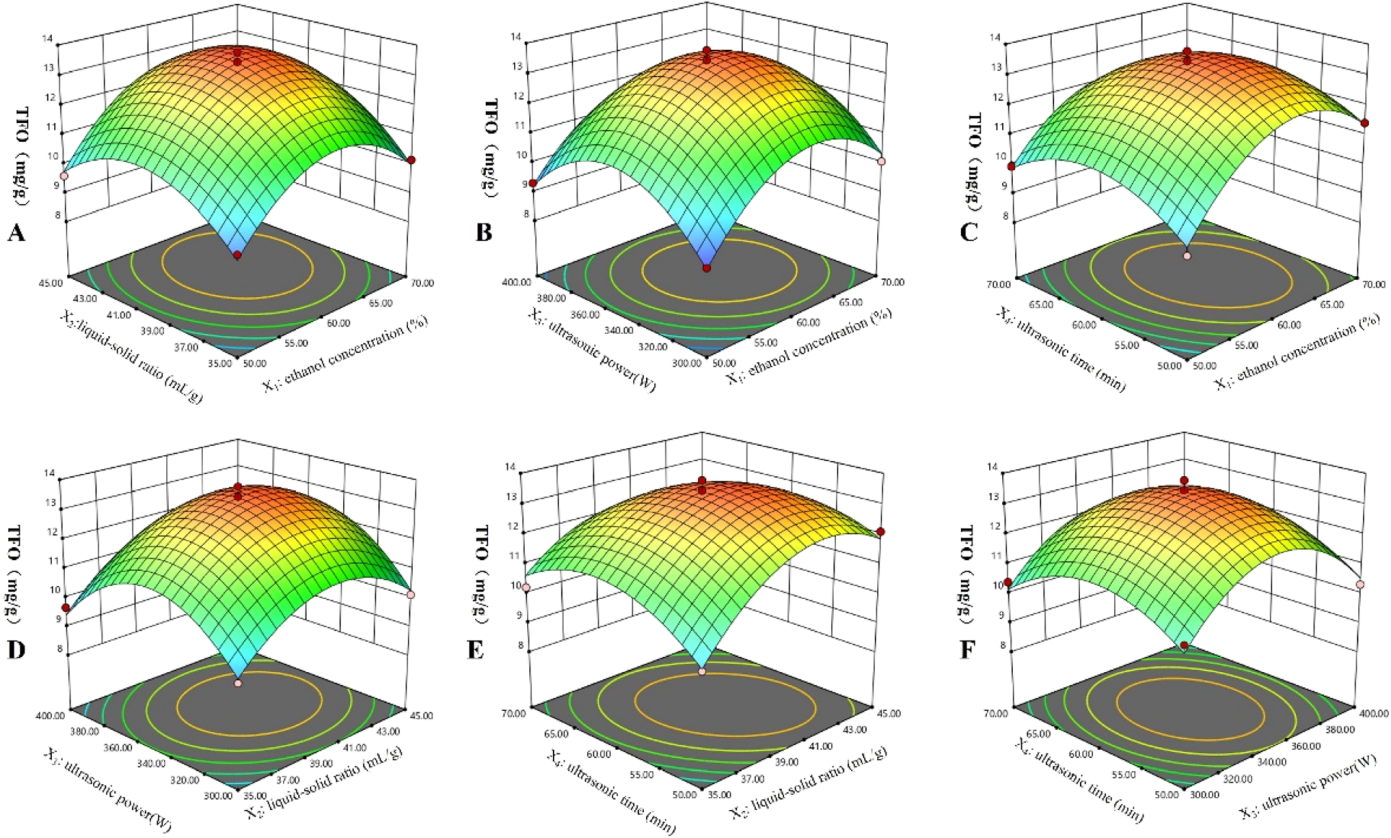
Response surface 3D plots. (A) The interaction effects of ethanol concentration and liquid–solid ratio; (B) the interaction effects of ethanol concentration and ultrasonic power; (C) the interaction effects of ethanol concentration and ultrasonic time; (D) the interaction effects of liquid–solid ratio and ultrasonic power; (E) the interaction effects of liquid–solid ratio and ultrasonic time; (F) the interaction effects of ultrasonic power and ultrasonic time.

The 3D graphical representations (illustrated in Fig. 4) elucidate the interactive influences exerted by the independent variables upon the yields of TFO. A steeper incline within these visual depictions signifies a more pronounced interaction between the paired factors, thereby highlighting their collective potency in modulating the response variable.^39^ Analysis revealed that the Prob > *F* values for the interactions involving *X*_1_*X*_2_, *X*_1_*X*_3_, *X*_1_*X*_4_, *X*_2_*X*_3_ and *X*_3_*X*_4_ were all above 0.05, which implies insignificant interaction effects on the ultrasonic extraction rate; on the other hand, the interaction term *X*_2_*X*_4_ had a Prob > *F* value lower than 0.01, indicating a highly significant interaction effect. The optimal extraction parameters ascertained through RSM were delineated as follows: an ethanol concentration of 61.95%, a liquid–solid ratio of 41.06 mL g^−1^, an ultrasonic power setting of 351.57 W, and an ultrasonic exposure duration of 58.95 minutes, yielding a TFO concentration of 13.538 mg g^−1^. In consideration of practical experimental constraints, these conditions were judiciously adjusted for the validation experiment, resulting in an ethanol concentration of 62%, a liquid–solid ratio of 41 mL g^−1^, an ultrasonic system power of 350 W, and an ultrasonic treatment interval of 59 minutes.

### 2.5. Optimization of extraction parameters utilizing RSM-ANN-GA

Utilizing the dataset derived from RSM analyses, a Back Propagation (BP) Artificial Neural Network (ANN) was trained, incorporating four pivotal factors-ethanol concentration, liquid–solid ratio, ultrasonic power and ultrasonic time-as input parameters, with the extraction yield of TFO serving as the output metric. The architectural topology of this artificial neural network is visually represented in Fig. S1.† The hyperbolic tangent sigmoid (“tansig”) function was utilized as the activation function bridging the input layer and the hidden layer, whereas the linear (“purelin”) function served as the transfer mechanism linking the hidden layer to the output layer. Employing the Levenberg–Marquardt backpropagation (“trainlm”) algorithm as the training function, the network was meticulously calibrated with a regimen encompassing 1000 epochs, 25 iterations per epoch, and a training goal error of 0.0001. Consequently, a network topology featuring 4 input nodes, 10 hidden nodes, and a solitary output node was architecturally realized, as depicted in Fig. S1.† As exemplified in Fig. S2† and 5, the best validation performance was 0.32438 at epoch 6 and the correlation coefficients pertaining to the training data, validation data, test data, and the aggregate dataset were recorded at 0.99916, 0.96762, 0.94672, and 0.93688, respectively. Notably, the correlation coefficients across all four sample categories surpassed the benchmark of 0.90, a testament to the commendable fitting capacity of the BP-ANN model for training, validation, and testing samples alike. In essence, the constructed BP-ANN model manifests potent predictive potential with regard to the test outcomes.

**Fig. 5:**
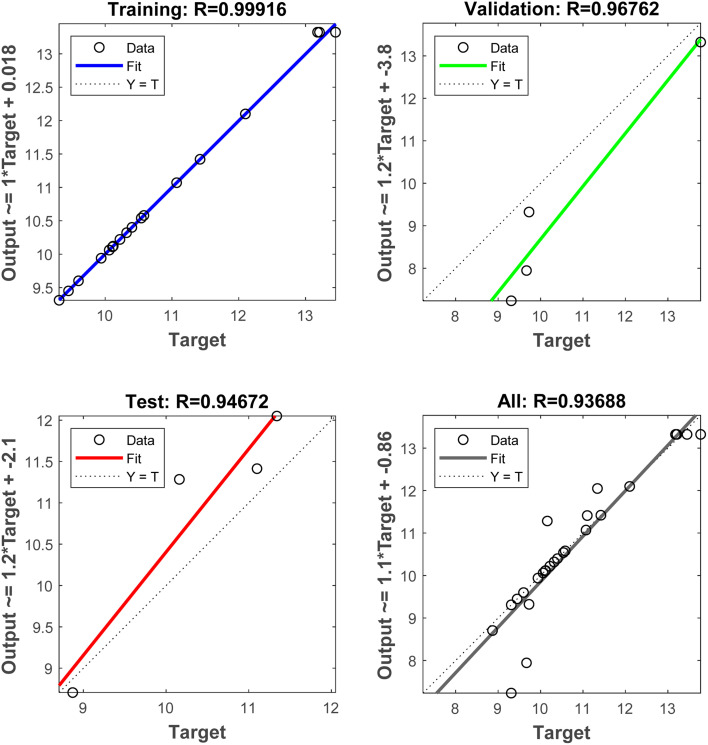
Training, validation, testing and fit of all data to the BP-ANN simulation output values.

To optimize the output maximization, the network data emanating from the RSM-ANN-GA integration was adopted as the objective function. The quartet of independent variables was conceptualized as matrix variables, subject to boundary constraints, as delineated hereafter:[50; 35; 300; 50] ≤ [*X*_1_; *X*_2_; *X*_3_; *X*_4_] ≤ [70; 45; 400; 70]

The optimal extraction parameters, as determined through the sophisticated amalgamation of RSM-ANN-GA, were delineated as follows: an ethanol concentration of 58.93%, a liquid–solid ratio of 41.16 mL g^−1^, an ultrasonic power setting of 350.22 W, and an ultrasonic treatment duration of 58.18 minutes, culminating in a TFO concentration of 13.7844 mg g^−1^. In deference to the practicalities of experimental execution, these conditions were judiciously refined for the validation phase, resulting in an ethanol concentration of 59%, a liquid–solid ratio of 41 mL g^−1^, an ultrasonic power output of 350 W, and an ultrasonic treatment interval of 58 minutes.

### 2.6. Validation experiment

In accordance with the optimal extraction parameters ascertained *via* RSM and the integrated RSM-ANN-GA approach, a trio of replicate experiments was meticulously conducted for each method. Subsequently, the relative errors were computed to critically assess the predictive efficacy of the respective methodologies. As evidenced by the tabulated data in Table 2, the prediction outcome of the RSM-ANN-GA integration exhibited a notably reduced relative error, accompanied by a superior extraction yield compared to the standalone RSM technique.

**Table 2:** Validation experiments of the optimal extraction conditions by RSM and RSM-ANN-GA

Method	The optimal extraction conditions				Result (TFO)		
	Ethanol concentration (*X*_1_)	Liquid–solid ratio (*X*_2_)	Ultrasonic power (*X*_3_)	Ultrasonic time (*X*_4_)	Experiment	Predicted	RE (%)
RSM	62	41	350	59	13.3533 ± 0.0241	13.538	−1.36 ± 0.18
RSM-ANN-GA	59	41	350	58	13.6608 ± 0.0251	13.7844	−0.90 ± 0.18

### 2.7. Antioxidant activity of TFO

Under physiological homeostasis, the generation and scavenging of free radicals within the body maintain a delicate equilibrium. However, an overabundance of free radicals can precipitate a redox imbalance, disrupting the organism's metabolic functions and culminating in the manifestation of disease states. This perturbation of the body's natural redox equilibrium, triggered by an excess of free radicals, leads to oxidative stress, which is implicated in the pathogenesis of numerous maladies.

Extensive scholarly investigations have unequivocally demonstrated that an array of antioxidant enzymes inherent in the human body possess the capability to efficaciously neutralize free radicals.^40–42^ However, concomitant with the progression of chronological age, there ensues a decrement in the activity of these antioxidant enzymes, thereby attenuating the organism's proficiency in scavenging free radicals. This decline precipitates an accumulation of free radicals, a concomitant elevation in the concentration of malondialdehyde-a marker of oxidative stress-and an exacerbation of the extent of oxidative damage inflicted upon the body. Such a cascade of events culminates in the onset and progression of senescence.^43^

**Fig. 6:**
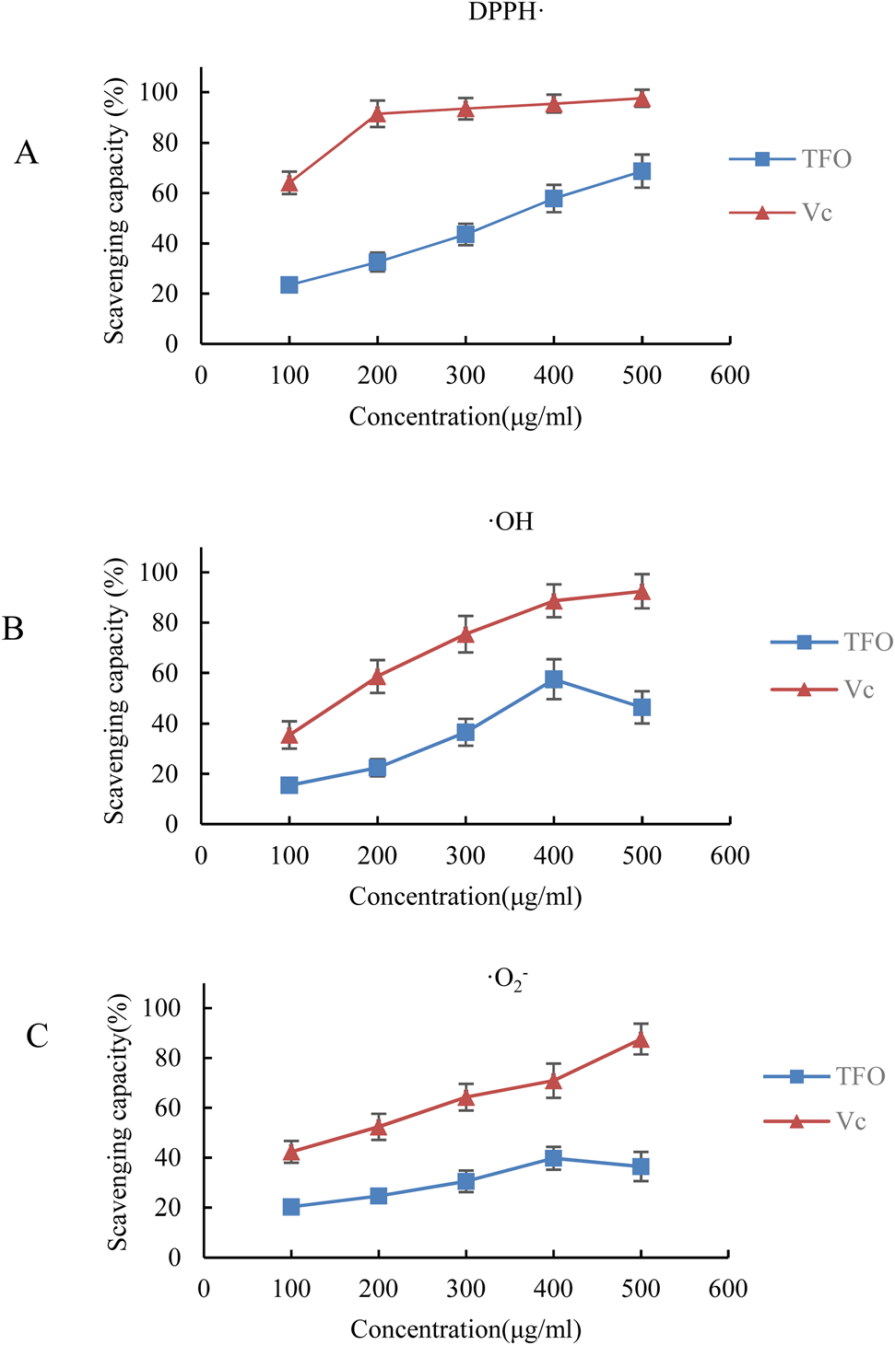
The antioxidant capacity of TFO *in vitro*. (A) The scavenging capacity on DPPH free radical (DPPH˙); (B) the scavenging capacity on hydroxyl free radical (˙OH); (C) the scavenging capacity on superoxide anion free radical (˙O_2_^−^).

To ascertain the antioxidant efficacy of TFO, a series of *in vitro* assays were meticulously conducted within the ambit of this investigation. As illustrated in Fig. 6, TFO demonstrated a pronounced propensity to scavenge DPPH, hydroxyl, and superoxide anion radicals (DPPH˙, ˙OH and ˙O_2_^−^, respectively), albeit exhibiting a less pronounced effect compared to vitamin C (V_C_) when administered at equivalent concentrations.

## 3 Experimental section

### 3.1. Materials

*Oxalis corniculata*, sourced from the Lushan Mountain Nature Reserve (situated between 115°52′ and 116°08′ east longitude and 29°26′ to 29°41′ north latitude) in Jiangxi Province, was taxonomically authenticated by Dr Keyue Liu from the School of Pharmacy and Life Sciences at Jiujiang University, Jiujiang, China. The plant material underwent a controlled drying process at 60 °C for a duration of 12 hours (water content less than 5%), post which it was finely ground and subsequently sieved through a 40 mesh sieve. Thereafter, the processed herbs were meticulously stored under cool conditions to preserve their integrity and pharmacological properties.

### 3.2. Chemicals and reagents

The 1,1-diphenyl-2-picrylhydrazyl (DPPH) reagent (lot: 8R7RL-JT, purity > 97%) was acquired from Tokyo Chemical Industry Development Co., Ltd., based in Shanghai, China. Rutin (lot: M1013A, purity > 97%) was obtained from Dalian Meilun Biotech Co., Ltd., headquartered in Dalian, China. For Fourier transform infrared (FTIR) spectroscopic analysis, potassium bromide of spectral purity grade was sourced from Tianjin Kemiou Chemical Reagent Co., Ltd., situated in Tianjin, China. Ultra-pure water utilized in the experimental procedures was produced using a Milli-Q purification system. All other chemical reagents and solvents employed throughout this investigation were of analytical grade, ensuring the highest quality standards for accurate and reliable results.

### 3.3. Experimental design

#### 3.3.1. Quantitative determination of flavonoid constituents in extracted samples

The quantification of flavonoids was achieved through the employment of the NaNO_2_–Al(NO_3_)_3_–NaOH spectrophotometric assay, with rutin serving as the standard compound.^35^ Precisely 30 mg of rutin standard was accurately weighed and dissolved in 60% ethanol, followed by heating in a water bath maintained at 38 °C until fully dissolved. Upon cooling, the solution was transferred to a 100 mL volumetric flask and diluted to the mark with 60% ethanol. A reference solution, with a concentration of 0.3 mg mL^−1^, was thus prepared and stored at 4 °C in the refrigerator to ensure stability and prevent degradation.

With meticulous precision, volumes of 0.8, 1.0, 1.2, 1.4, 1.6, 1.8, and 2.0 milliliters of the rutin standard solution were aliquoted. Each aliquot was then supplemented with 0.3 mL of a 5% sodium nitrite solution, vigorously shaken, and allowed to rest for a period of 6 minutes. Subsequently, 0.3 mL of a 10% aluminum nitrate solution was added to each sample, followed by another round of thorough mixing and a subsequent resting phase of 6 minutes. Thereafter, 4 mL of a 0.1 mol per L sodium hydroxide solution was introduced. The solutions were adjusted to a final volume of 10 mL with 60% ethanol, shaken rigorously, and left to equilibrate for a 10 minute interval. Spectral scans in the UV-VIS range from 400 to 600 nm were performed on the rutin standard solutions. The absorption peak at 505.2 nm was selected for quantitative analysis. A linear regression equation was established by plotting the concentration of the standard solution (mg mL^−1^) against the absorbance readings.

The extraction of TFO was facilitated through an ultrasonic-assisted extraction (UAE) technique. Precisely 1.00 g of the powdered plant material was weighed and subjected to extraction with a predetermined volume of ethanol. The mixture was exposed to ultrasonic agitation for a specified duration, followed by filtration to separate the solid residue from the liquid extract. Ethanol was then added to the filtrate to achieve a fixed volume of 50.00 mL. The total flavonoid content of the extract was assessed following the NaNO_2_–Al(NO_3_)_3_–NaOH colorimetric assay protocol. The concentration of TFO was subsequently quantified by applying the linear regression equation derived from the calibration curve.

#### 3.3.2. Identification and characterization of TFO

The identification of TFO was confirmed through the magnesium hydrochloride powder reaction assay. A minute quantity of magnesium powder was introduced to 5 mL of the extraction solution, followed by the addition of 5–10 drops of concentrated hydrochloric acid. The ensuing color alteration was meticulously observed and recorded. A predetermined amount of TFO was mixed with potassium bromide and scanned within the wavenumber range from 4000 to 400 cm^−1^ with 128 scans using an FTIR spectrometer (VERTEX 70, BRUKER, Germany). The blank (KBr pellet without test samples) used under the same settings was reported as reference spectra. The data were processed using OPUS 8.2 software (OPUS Series, BRUKER, Germany) following the collection of all spectra.

#### 3.3.3. Single factor experiment

To comprehensively evaluate the influence of diverse parameters on the yield of TFO, a series of single-factor experiments were meticulously designed. The ethanol concentration was varied between 40% and 80% (in steps of 10%), ultrasonic power was adjusted from 250 to 450 W (in increments of 50 W), the liquid–solid ratio was altered from 15 to 45 mL g^−1^ (in steps of 5 mL g^−1^), and the ultrasonic time ranged from 30 to 80 minutes (in intervals of 10 minutes).These variables were systematically manipulated to discern their individual impacts on the extraction efficiency of TFO.

#### 3.3.4. The RSM modeling

The statistical software package Design-Expert 13.0 (Minneapolis, MN, USA) was enlisted to construct RSM models. Drawing upon the outcomes of preliminary single-factor experimentation, a Box–Behnken Design (BBD) was meticulously crafted, incorporating four independent variables and three levels for each variable. The levels of these factors were coded as −1, 0, and 1, as delineated in Table 1. A comprehensive series of 29 experimental trials were executed, the outcomes of which are tabulated in Table 2. The gathered data were subjected to quadratic polynomial modeling, yielding a predictive mathematical equation that elucidates the interactions and effects of the variables on the response:^22^1
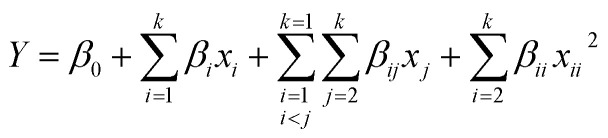


Within the quadratic model equation, the parameters *β*_0_, *β*_*i*_, *β*_*ii*_, and *β*_*ij*_ represent the regression coefficients, where *β*_0_ signifies the intercept, *β*_*i*_ denotes the linear effect coefficients, *β*_*ii*_ corresponds to the squared term coefficients indicating the curvature of the response surface, and *β*_*ij*_ represents the interaction effect coefficients. Here, *x*_*i*_ and *x*_*j*_ denote the coded levels of the independent variables that exert influence over the dependent response variable *Y*, while *k* signifies the total number of variables under consideration.

Analysis of Variance (ANOVA) was employed to scrutinize the RSM fitting model, thereby evaluating the statistical significance of each constituent term within the model. This rigorous statistical test facilitated the determination of whether the variations in the response variable could be attributed to the independent variables or were simply due to random error.

#### 3.3.5. RSM-ANN-GA modeling

Employing the Neural Network Toolbox™ integrated within MATLAB R2019a, a sophisticated ANN model was constructed utilizing the Genetic Algorithm (GA) for optimization. Drawing from the dataset generated through RSM, the BP-ANN was meticulously trained. The training regimen involved four critical input variables: ethanol concentration, liquid–solid ratio, ultrasonic power, and ultrasonic time. These inputs were directed towards predicting the output variable, specifically the extraction yield of TFO.

**Fig. 7:**
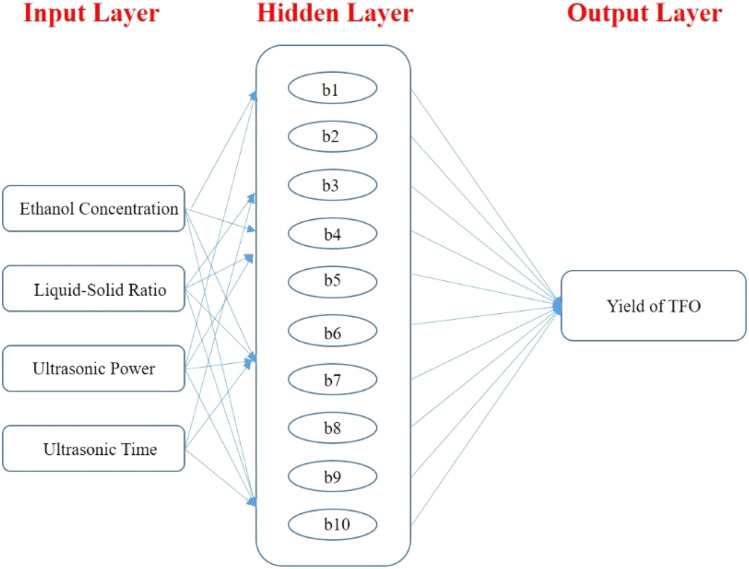
Structure diagram of BP neural network model.

Guided by the minimization of the Mean Square Error (MSE) function, a 4-10-1 neural network model architecture was meticulously designed, as depicted in Fig. 7. In the construction of this network, the Levenberg–Marquardt algorithm was selected for its efficiency in minimizing the error. Of the total RSM samples, 70% (comprising 21 data points) were allocated for the training phase to teach the network the underlying patterns in the data. An additional 15% (equivalent to 4 points) were reserved for validation purposes, ensuring the network's generalizability beyond the training dataset. Similarly, another 15% (also 4 points) were set aside for testing the network's performance on unseen data. The optimal ANN model was identified based on the lowest MSE value and the highest correlation coefficient (*R* value). GA optimization, encompassing operations like reproduction, crossover, and mutation, was subsequently employed to fine-tune the model parameters for enhanced performance.^25^

#### 3.3.6. Confirmatory experiment

The samples were accurately weighed, and three parallel experiments were carried out according to the optimal extraction parameters predicted by RSM and RSM-ANN-GA. The relative errors of the actual and predicted extraction rates of TFO were calculated.

With meticulous precision, the samples were weighed, and a series of triplicate experiments were meticulously executed in strict adherence to the optimal extraction parameters forecasted by both the RSM and the RSM-ANN-GA models. The discrepancies between the actual extraction yields of TFO and those predicted by the models were quantified as relative errors, offering a measure of the models' predictive accuracy.

### 3.4. Antioxidative study of TFO *in vitro*

Pursuant to the delineated optimal extraction protocols, the isolation of TFO was successfully executed. Upon completion of the evaporation process, the concentration of flavonoids was quantified at an impressive 76.8%. The antioxidant potential of the extracted TFO was subsequently appraised through the measurement of its ability to scavenge DPPH radicals (DPPH˙), hydroxyl radicals (˙OH), and superoxide anions (˙O_2_^−^). This evaluation was contextualized against vitamin C (V_C_), a widely recognized antioxidant, which served as the benchmark standard in assessing the comparative efficacy of TFO's free-radical scavenging capabilities.

#### 3.4.1. Scavenging effect on DPPH˙

Solutions of TFO and vitamin C (V_C_) were meticulously prepared across a concentration gradient of 100, 200, 300, 400 and 500 μg mL^−1^. These solutions were then subjected to thorough mixing to ensure complete homogeneity prior to analysis. A 0.15 mmol L^−1^ solution of 1,1-diphenyl-2-picrylhydrazyl (DPPH) was concurrently prepared using an 80% ethanol solvent. Subsequently, 0.11 mL of the DPPH solution was judiciously combined with 0.01 mL aliquots of each sample concentration, with immediate mixing to facilitate the onset of the reaction. These mixtures were allowed to incubate in the dark at room temperature for a period of thirty minutes to permit the radical scavenging process to occur. Absorbance readings of each sample were then acquired at a wavelength of 517 nm utilizing a spectrophotometer, from which the percentage clearance rate was calculated as a measure of antioxidant capacity:^44–46^2
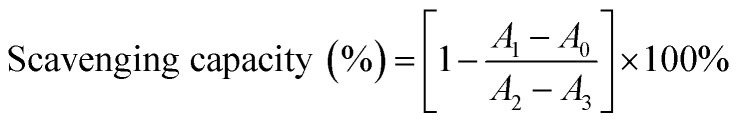
*A*_1_: the absorbance of DPPH solution mixed with the sample solution, *A*_0_: the absorbance of the sample solution, *A*_2_: the absorbance of DPPH solution, *A*_3_: the absorbance of 80% ethanol solution.

#### 3.4.2. Scavenging effect on ˙OH

A volumetric pipette was used to carefully dispense 0.2 mL of a 0.75 mmol per L *o*-diazepine solution into a clean, pre-chilled Eppendorf tube. Subsequently, 0.4 mL of a 0.2 mol per L phosphate buffer solution (PBS), precisely adjusted to pH 7.40, was added to the aforementioned solution to maintain optimal pH conditions for the ensuing reaction. Next, 0.2 mL of a 0.75 mmol per L FeSO_4_ solution was introduced to serve as a catalyst in the formation of hydroxyl radicals *via* the Fenton reaction. Then, 0.2 mL of the sample solution, containing TFO or V_C_ at varying concentrations, was precisely added to the mixture to assess its hydroxyl radical scavenging capacity. Finally, 0.2 mL of a 0.01% hydrogen peroxide (H_2_O_2_) solution was added to trigger the radical formation. The entire reaction mixture was then incubated in a water bath at a temperature of 37 °C for a duration of 60 minutes to ensure adequate time for the reaction to occur. Following the incubation period, the absorbance of each sample was accurately measured at a wavelength of 536 nm using a high-performance spectrophotometer. The clearance rate was subsequently calculated, providing a quantitative measure of the antioxidant efficacy of the tested samples against hydroxyl radicals:^47^3
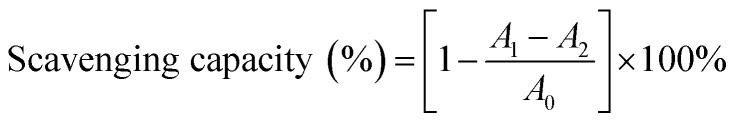
*A*_1_: the absorbance of the sample solution mixed with *o*-diazepine, FeSO_4_ and H_2_O_2_, *A*_2_: the absorbance of the sample solution with FeSO_4_ and H_2_O_2_, *A*_0_: the absorbance of *o*-diazepine, FeSO_4_ and H_2_O_2_ solution.

#### 3.4.3. Scavenging effect on ˙O_2_^−^

Precisely measure 1.0 mL of the sample solution at various concentrations and gently introduce it into a volumetric flask containing 5.00 mL of a phosphoric acid buffer solution, accurately adjusted to a pH of 8.2. This mixture was then subjected to a preheating process in a water bath maintained at a constant temperature of 25 °C for a duration of 20 minutes, ensuring thermal equilibrium. Subsequently, 1.0 mL of a preheated pyrogallol solution (4.50 mmol L^−1^) was swiftly added to the mixture, followed by thorough blending to promote homogeneity. The concoction was then returned to the water bath, where it was held at 25 °C for an additional 4 minutes to facilitate the reaction. To terminate the reaction instantaneously, 1 mL of HCl (8.00 mmol L^−1^) was added to the mixture. The absorbance of the solution was then determined at a wavelength of 320 nm using a spectrophotometer of high sensitivity. From these absorbance values, the clearance rate was calculated, providing a quantitative assessment of the sample's ability to scavenge radicals:^48^4
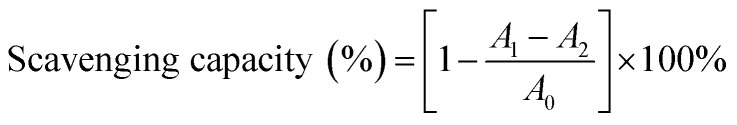
*A*_1_: the absorbance of pyrogallic acid mixed with the sample solution, *A*_2_: the absorbance of the sample solution, *A*_0_: the absorbance of pyrogallic acid.

### 3.5. Statistical analysis

The outcomes of experiments were articulated as the mean and standard deviation (mean ± SD). The Design-Expert Software 13.0 and Neural Network Toolbox™ in MATLAB R2019a were used for RSM and RSM-ANN-GA analysis, respectively.

## 4 Conclusions

This comprehensive study aims to optimize the UAE process for the efficient extraction of TFO. The optimization strategy builds upon the RSM by further employing ANN and GA to obtain the optimal process conditions. This method combines the advantages of RSM, ANN, and GA, not only enhancing the precision of the extraction parameters but also highlighting the superiority of the RSM-ANN-GA hybrid model in terms of predictive accuracy and extraction efficiency.

Additionally, the study delves into the antioxidant properties of the extracted total flavonoids, providing critical insights into their potential health benefits. Through rigorous *in vitro* testing, the antioxidant activities of the flavonoids were assessed, revealing their effectiveness in scavenging reactive oxygen species such as DPPH, hydroxyl, and superoxide anions. This research not only contributes to the advancement of extraction techniques but also highlights the therapeutic potential of *O. corniculata* as a rich source of natural antioxidants.

In general, in consonance with a plethora of preceding investigations, the ANN emerges as a remarkably potent mathematical construct, adept at both optimizing and predicting the intricacies of the extraction process.^25,27,30^ This finding underscores the considerable potential of this methodology for broader application within the domain of traditional Chinese medicine, particularly concerning the extraction of its pharmacologically active constituents. Given its effectiveness in enhancing process efficiency and predictive accuracy, utilizing artificial neural networks to optimize extraction methods such as enzyme-assisted extraction (EAE), microwave-assisted extraction (MAE) and deep eutectic solvent extraction (DESE) represents a promising avenue for future research and practical applications.

In recent years, alongside ultrasonic-assisted extraction (UAE), a variety of innovative extraction techniques including supercritical fluid extraction (SCFE),^49^ subcritical water extraction (SWE),^50^ hot pressurized liquid extraction (HPLE),^51^ microwave-assisted extraction (MAE),^52^ high hydrostatic pressure extraction (HHPE),^53^ pulsed electric field (PEF) extraction,^54^ and high-voltage electrical discharge (HVED)^55^ extraction have gained prominence in the field of plant component extraction. Additionally, hybrid methodologies such as SCFE coupled with pressurized liquid extraction (SCFE-PLE),^56^ integration of HHPE with UAE (HHPE-UAE),^57^ synergistic application of PEF with UAE (PEF-UAE),^58^ and the combination of HVED with UAE (HVED-UAE)^57^ have emerged. Therefore, adopting innovative extraction techniques, particularly utilizing the synergistic effects of combined methods, and integrating ANN for process optimization, can help enhance extraction efficiency and improve the quality of extracted products, making it worthy of further exploration in future research.

## Abbreviations

TFO: Total flavonoids from *Oxalis corniculata*
RSM: Response surface methodology
ANN: Artificial neural networks
GA: Genetic algorithms
UAE: Ultrasound-assisted extraction
OVAT: One-variable-at-a-time
DoE: Design of experiments
FTIR: Fourier-transform infrared
BP-ANN: Back propagation artificial neural network
DPPH: 1,1-Diphenyl-2-picrylhydrazyl
BBD: Box–Behnken design
ANOVA: Analysis of variance
MSE: Mean square error
DPPH˙: DPPH radicals
˙OH: Hydroxyl radicals
˙O_2_^−^: Superoxide anions
EAE: Enzyme-assisted extraction
DESE: Deep eutectic solvent extraction
SCFE: Supercritical fluid extraction
SWE: Subcritical water extraction
HPLE: Hot pressurized liquid extraction
MAE: Microwave-assisted extraction
HHPE: High hydrostatic pressure extraction
PEF: Pulsed electric field

## Data availability

Data are contained within the article or ESI.†

## Author contributions

Conceptualization, D.-Z. J. and K.-Y. L.; methodology, D.-P. Y.; data analysis, M. Z. and W.-B. L.; resources, K.-Y. L.; FTIR spectrum analysis, D.-L. L. and M. Z.; investigation, M. Z. and D.-Z. J.; writing—original draft preparation, D.-P. Y.; writing—review and editing, supervision, D.-Z. J.; funding acquisition, D.-Z. J. All authors have read and agreed to the published version of the manuscript.

## Conflicts of interest

The authors declare no conflict of interest.

## Supplementary Material

RA-014-D4RA05077K-s001
