# Supplementary material for: Optimization of ultrasonic-assisted extraction of total flavonoids from Oxalis corniculata by a hybrid response surface methodology-artificial neural network-genetic algorithm (RSM-ANN-GA) approach, coupled with an assessment of antioxidant activities
Source: RSC Adv. 2024 Dec 10;14(52):39069–80. doi: 10.1039/d4ra05077k (PMC11629873; doi:10.1039/d4ra05077k)
Supplement: RA-014-D4RA05077K-s001 [file RA-014-D4RA05077K-s001.pdf]

Supplementary Information (SI) for RSC Advances.
This journal is © The Royal Society of Chemistry 2024

Supplementary data

Optimization of Ultrasonic-Assisted Extraction of Total Flavonoids from *Oxalis corniculata* by a Hybrid Response Surface Methodology-Artificial Neural Network-Genetic Algorithm (RSM-ANN-GA) Approach, Coupled with an Assessment of Antioxidant Activities

Tab. S1 Factors and levels in Box–Behnken design

Level	Factor			
	ethanol concentration(X_1)	liquid-solid ratio(X_2)	ultrasonic power (X_3)	ultrasonic time (X_4)
1	70%	45mL/g	400w	70min
0	60%	40 mL/g	350w	60min
-1	50%	35 mL/g	300w	50min

Tab. S2 Box-Behnken design with RSM and ANN results

Std	X_1	X_2	X_3	X_4	TFO yield (mg/g)		
					Experiment	RSM Predicted	ANN Predicted
1	70	40	400	60	10.11±0.06	10.31	10.10
2	50	40	350	70	9.94±0.05	9.87	10.06
3	60	40	350	60	13.21±0.13	13.41	13.42
4	60	45	300	60	10.12±0.08	10.27	10.34
5	50	40	400	60	9.31±0.06	9.20	9.22
6	60	40	350	60	13.45±0.12	13.41	13.42
7	50	40	350	50	9.31±0.06	9.57	9.20
8	60	40	350	60	13.18±0.18	13.41	13.42
9	70	45	350	60	11.34±0.07	11.48	11.34
10	60	35	350	50	9.73±0.05	9.80	9.67
11	60	40	400	50	10.32±0.07	10.53	10.28
12	60	40	350	60	13.45±0.11	13.41	13.42
13	60	40	300	70	10.40±0.06	10.15	10.40
14	60	40	350	60	13.77±0.16	13.41	13.62
15	60	45	350	50	12.10±0.11	11.86	12.07
16	60	40	300	50	10.54±0.05	10.29	10.50
17	60	40	400	70	10.06±0.06	10.28	10.02
18	50	45	350	60	9.60±0.05	9.74	9.62
19	50	35	350	60	9.31±0.06	9.13	9.30

20	60	35	400	60	9.67±0.07	9.40	9.52
21	70	35	350	60	10.16±0.05	9.99	10.16
22	60	35	350	70	10.22±0.08	10.62	10.13
23	70	40	300	60	10.06±0.05	10.32	10.05
24	60	45	400	60	11.10±0.06	10.83	10.71
25	70	40	350	50	11.42±0.10	11.37	11.41
26	70	40	350	70	11.07±0.07	10.69	11.07
27	50	40	300	60	8.87±0.05	8.82	8.87
28	60	45	350	70	10.58±0.08	10.66	10.54
29	60	35	300	60	9.45±0.06	9.60	9.47

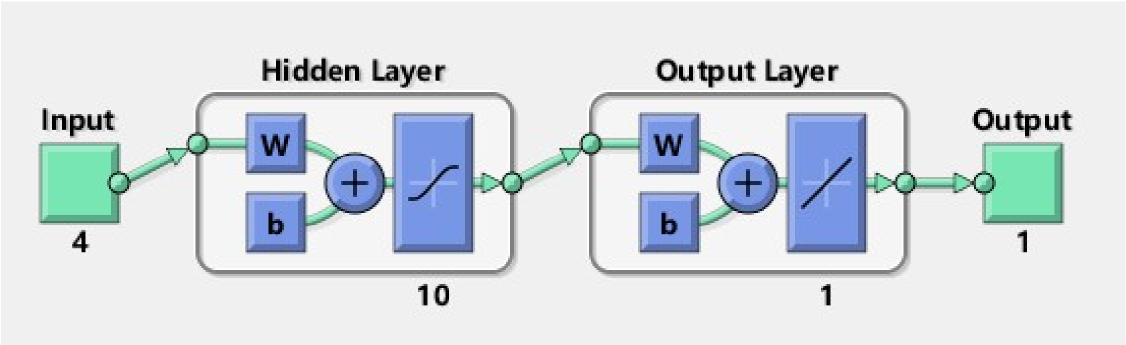

Fig. S1 The artificial neural network architecture topology

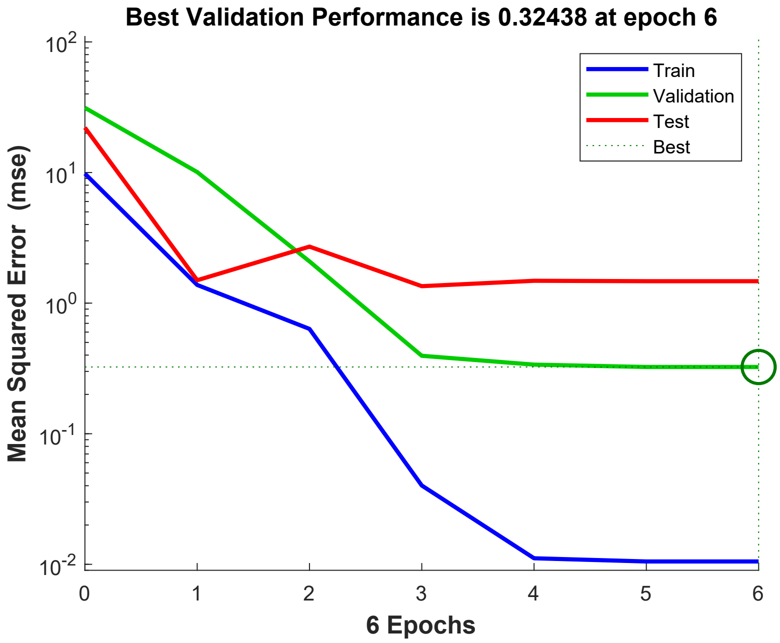

Fig. S2 BP-ANN training performance of TFO
